# Micromechanics of Stress-Softening and Hysteresis of Filler Reinforced Elastomers with Applications to Thermo-Oxidative Aging

**DOI:** 10.3390/polym12061350

**Published:** 2020-06-15

**Authors:** Jan Plagge, Manfred Klüppel

**Affiliations:** Deutsches Institut für Kautschuktechnologie e. V., Eupener Str. 33, D-30519 Hannover, Germany; jan.plagge@dikautschuk.de

**Keywords:** filled elastomers, stress softening, filler-induced hysteresis, cluster mechanics, FEM simulation

## Abstract

A micromechanical concept of filler-induced stress-softening and hysteresis is established that describes the complex quasi-static deformation behavior of filler reinforced rubbers upon repeated stretching with increasing amplitude. It is based on a non-affine tube model of rubber elasticity and a distinct deformation and fracture mechanics of filler clusters in the stress field of the rubber matrix. For the description of the clusters we refer to a three-dimensional generalization of the Kantor–Webman model of flexible chain aggregates with distinct bending–twisting and tension deformation of bonds. The bending–twisting deformation dominates the elasticity of filler clusters in elastomers while the tension deformation is assumed to be mainly responsible for fracture. The cluster mechanics is described in detail in the theoretical section, whereby two different fracture criteria of filler–filler bonds are considered, denoted “monodisperse” and “hierarchical” bond fracture mechanism. Both concepts are compared in the experimental section, where stress–strain cycles of a series of ethylene–propylene–diene rubber (EPDM) composites with various thermo-oxidative aging histories are evaluated. It is found that the “hierarchical” bond fracture mechanism delivers better fits and more stable fitting parameters, though the evolution of fitting parameters with aging time is similar for both models. From the adaptations it is concluded that the crosslinking density remains almost constant, indicating that the sulfur bridges in EPDM networks are mono-sulfidic, and hence, quite stable—even at 130 °C aging temperature. The hardening of the composites with increasing aging time is mainly attributed to the relaxation of filler–filler bonds, which results in an increased stiffness and strength of the bonds. Finally, a frame-independent simplified version of the stress-softening model is proposed that allows for an easy implementation into numerical codes for fast FEM simulations

## 1. Introduction

Nanoscopic fillers like carbon black or silica play an important role in the mechanical reinforcement of elastomers [1,2]. They make the elastomer stiffer and tougher, leading to a pronounced reduction of crack propagation rates and wear, which is accompanied by an increased life time of rubber goods [3]. But the incorporation of fillers results in a nonlinear dynamic-mechanical response, which is reflected e.g., by the amplitude-dependence of the dynamic moduli. This so called Payne effect was investigated by several authors like Payne [4] and Medalia [5]. A related phenomenon is the stress softening effect under quasi-static cyclic deformation, which is also called Mullins effect due to the intensive studies by Mullins [6]. Accordingly, a drop in stress appears if the loading goes beyond the previous maximum. Most of the stress drop at a certain strain occurs in the first cycle, and in the following cycles the specimen approaches a steady state stress–strain curve. A second characteristic effect caused by fillers is the pronounced hysteresis which is related to the dissipation of mechanical energy during every cycle.

Based on the investigations of filler network morphology by different experimental techniques, a model of rubber reinforcement by flexible filler clusters has been proposed that allows for a quantitative understanding of the complex mechanical response under quasi-static and dynamic deformations [7,8,9,10]. This model of rubber reinforcement refers to the kinetics of cluster–cluster aggregation (CCA) of filler networking in elastomers, which represents a reasonable theoretical basis for analyzing the linear viscoelastic properties of reinforced rubbers. According to this approach, filler networks consist of a space-filling configuration of fractal CCA-clusters with characteristic mass fractal dimension *df* ≈ 1.8. The mechanical response of such filler networks at small strain depends purely on the fractal connectivity of the CCA-clusters. It can be evaluated by referring to the Kantor–Webman model [11] of flexible chains of filler particles that allows for a micromechanical description of the elastic properties of tender CCA-clusters in elastomers. The main contribution of the elastically stored energy in the strained filler clusters results from the bending–twisting deformation of filler–filler bonds, considered by an elastic constant $\bar{G}$. Based on this approach a power law behavior of the small strain modulus vs. filler concentration can be derived. The predicted exponent 3.5 is in good agreement with experimental data of Payne [4] for carbon black filled butyl rubber. This power law behavior has been confirmed by further investigations of carbon black and silica filled rubbers as well as composites with polymeric model fillers (microgels) [7,8,9,10].

Based on this approach, a micromechanical material model of filler reinforced elastomers has been put forward, denoted dynamic flocculation model (DFM) [7,12,13,14,15,16]. Similar models have been proposed by other authors [17,18,19]. The DFM describes the complex quasi-static stress–strain response of filler reinforced elastomers up to large strains in fair agreement with experimental data. It is based on a non-affine tube model of rubber elasticity and considers hydrodynamic amplification of the rubber matrix by a fraction of hard rigid filler clusters with filler–filler bonds in the unbroken, virgin state. The filler-induced hysteresis is described by the cyclic breakdown and re-aggregation of the residual fraction of more soft filler clusters with already broken, but reformed filler–filler bonds. The difference between hard and soft filler–filler bonds arises due to the softening of glassy-like polymer bridges between adjacent filler particles [7,20,21,22]. If they break under stress the recovery to a virgin bond will take time and/or high temperatures, as applied during vulcanization [23]. A finite element implementation of the DFM was established [24] by referring to the concept of representative directions introduced by Ihlemann [25]. Thereby also temperature and time dependent effects have been considered [26].

It is the aim of this study to consider the underlying fracture mechanics of filler clusters entering the DFM more closely, whereby we will investigate two different fracture criteria for filler–filler bonds. The differences between both approaches will be evaluated by comparing fitting results obtained for stress–strain cycles of ethylene–propylene–diene monomer rubber (EPDM) composites with various thermo-oxidative aging histories, which will be analyzed by the variation of fitting parameters. This is in close relation to recent investigations of the structure–property relationships of silica/silane formulations in different rubber composites [27] delivering microscopic information about the material properties of the systems. Finally, we will introduce a frame-invariant version of the stress-softening part of the DFM that allows for an easy implementation into a finite element code for fast finite element (FEM) applications of the isotropic discontinuous damage effects in engineering rubber science.

## 2. Modeling Approach

### 2.1. Basic Assumptions of the Dynamic Flocculation Model

The (microscopic) free energy density of the dynamic flocculation model (DFM) consists of two contributions, which are weighted by the effective filler volume fraction $\Phi_{\mathrm{eff}}$:

(1) $$W\left(\varepsilon_{\mu }\right)=\left(1-\Phi_{\mathrm{eff}}\right)W_{R}\left(\varepsilon_{\mu }\right)+\Phi_{\mathrm{eff}}\;W_{A}\left(\varepsilon_{\mu }\right)$$

The first addend is the equilibrium energy density stored in the extensively strained rubber matrix, which includes hydrodynamic amplification by a fraction of rigid filler clusters. The second addend considers the energy stored in the strained soft filler clusters and is responsible for the filler-induced hysteresis. The symbol $\varepsilon_{\mu }$ is defined in this work as the macroscopic strain in direction μ. The rubber elastic part is modeled by the free energy density of the extended non-affine tube model [12,28]:

(2) $$W_{R}\left(\varepsilon_{\mu }\right)=\frac{G_{c}}{2}\left\{\frac{\left(\sum_{\mu =1}^{3}\lambda_{\mu }^{2}-3\right)\left(1-\frac{T_{e}}{n_{e}}\right)}{1-\frac{T_{e}}{n_{e}}\left(\sum_{\mu =1}^{3}\lambda_{\mu }^{2}-3\right)}+\ln\left[1-\frac{T_{e}}{n_{e}}\left(\overset{3}{\underset{\mu =1}{\sum }}\lambda_{\mu }^{2}-3\right)\right]\right\}+2G_{e}\left(\overset{3}{\underset{\mu =1}{\sum }}\lambda_{\mu }^{-1}-3\right)$$

with $n_{e}$ being the number of statistical chain segments between neighboring entanglements and $T_{e}$ is the trapping factor $(0<T_{e}<1$) characterizing the fraction of elastically active entanglements. We define $\lambda_{\mu }$ to be the microscopic strain ratio (or stretch) on the nanoscale in direction μ. Thus, for unfilled rubbers the usual relation $\lambda_{\mu }=1+\varepsilon_{\mu }$ holds. The first addend in Equation (2) considers the constraints due to interchain junctions, with an elastic modulus $G_{c}$ proportional to the density of network junctions. The second addend considers topological constraints in densely packed polymer networks, whereby $G_{e}$ is proportional to the entanglement density of the rubber. The parenthetical expression in the first addend considers the finite chain extensibility of polymer networks by referring to an approach of Edwards and Vilgis [29]. For the limiting case $n_{e}/T_{e}=\sum \lambda_{\mu }^{2}-3$ a singularity is obtained for the free energy density $W_{R}$, indicating the maximum extensibility of the network. This is reached when the chains between successive trapped entanglements are fully stretched out. In the limit $n_{e}\to \infty$ the original Gaussian formulation of the non-affine tube model, derived by Heinrich et al. [30] for infinite long chains, is recovered.

The presence of tightly bonded (virgin bonds) rigid filler clusters gives rise to hydrodynamic reinforcement of the rubber matrix. This is specified by the strain amplification factor X as proposed by Mullins and Tobin [6], which relates the external, macroscopic strain $\varepsilon_{\mu }$ of the sample to the internal, microscopic strain ratio $\lambda_{\mu }$ of the rubber matrix, $\lambda_{\mu }=1+X\left(\varepsilon_{\mu ,\max}\right)\varepsilon_{\mu }$. For strain amplified rubbers this strain has to be used in the free energy density Equation (2). The microscopic stress of the rubber matrix is then obtained by differentiation with respect to the internal strain $\lambda_{\mu }$:

(3) $$\sigma_{R,\;\nu }\equiv \frac{\partial W_{R}}{\partial \lambda_{\nu }}=G_{c}\overset{3}{\underset{\mu =1}{\sum }}\frac{\partial \lambda_{\mu }}{\partial \lambda_{\nu }}\lambda_{\mu }\left\{\frac{\left(1-\frac{T_{e}}{n_{e}}\right)}{{\left(1-\frac{T_{e}}{n_{e}}\left(\sum_{\mu =1}^{3}\lambda_{\mu }^{2}-3\right)\right)}^{2}}-\frac{\frac{T_{e}}{n_{e}}}{1-\frac{T_{e}}{n_{e}}\left(\sum_{\mu =1}^{3}\lambda_{\mu }^{2}-3\right)}\right\}-2G_{e}\overset{3}{\underset{\mu =1}{\sum }}\frac{\partial \lambda_{\mu }}{\partial \lambda_{\nu }}\lambda_{\mu }^{-2}$$

This is the microscopic stress between the filler clusters that can be identified with the macroscopically measured engineering stress (1. PK stress) in equilibrium. For uniaxial deformations ($\lambda_{2}=\lambda_{3}=\lambda_{1}^{-1/2}$ and $\partial \lambda_{2}=\partial \lambda_{3}=-1/2\;\lambda_{1}^{-3/2}\partial \lambda_{1}$) we obtain for the engineering stress in stretching direction:

(4) $$\sigma_{R,\;1}=G_{c}\left(\lambda_{1}-\lambda_{1}^{-2}\right)\left\{\frac{\left(1-\frac{T_{e}}{n_{e}}\right)}{{\left(1-\frac{T_{e}}{n_{e}}(\lambda_{1}^{2}+2/\lambda_{1}-3\right)}^{2}}-\frac{\frac{T_{e}}{n_{e}}}{1-\frac{T_{e}}{n_{e}}\left(\lambda_{1}^{2}+2/\lambda_{1}-3\right)}\right\}+2G_{e}\left(\lambda_{1}^{-\frac{1}{2}}-\lambda_{1}^{-2}\right)$$

In the case of a preconditioned sample and for strains smaller than the previous straining $\left(\varepsilon_{\mu }<\varepsilon_{\mu ,\max}\right)$, the materials microscopic structure is already adjusted to the maximum load and the strain amplification factor X is independent of strain. In that case it is determined by $\varepsilon_{\mu ,\max}$ ($X=X\left(\varepsilon_{\mu ,\max}\right))$. We relate this to the irreversible fracture of filler clusters (see below). A relation for the strain amplification factor of overlapping fractal clusters of size ξ was derived by Huber and Vilgis [31]. By using path integral methods they found $X=1+c\Phi^{2/\left(3-d_{f}\right)}\xi^{d_{w}-d_{f}}$ where Φ is the filler volume fraction and c is a constant of order one. With this, $X\left(\varepsilon_{\mu ,\max}\right)$ can be evaluated by averaging over the size distribution of hard clusters in all space directions. In the case of preconditioned samples this yields:

(5) $$X\left(\varepsilon_{\mu ,\max}\right)=1+c\Phi_{\mathrm{eff}}^{\frac{2}{3-d_{f}}}\overset{3}{\underset{\mu =1}{\sum }}\frac{1}{d}\left[\int_{0}^{\xi_{\mu ,\min}}{\left(\frac{\xi_{\mu }^{’}}{d}\right)}^{d_{w}-d_{f}}\varphi \left(\xi_{\mu }^{’}\right)d\xi_{\mu }^{’}+\int_{\xi_{\mu ,\min}}^{\infty }\varphi \left(\xi_{\mu }^{’}\right)d\xi_{\mu }^{’}\right]$$

Here, d is the particle size, $\xi_{\mu }$ is the cluster size in spatial direction μ and $\xi_{\mu ,\min}$ is the minimum cluster size which will be calculated later on. The fractal exponents are determined as $d_{f}\approx 1.8$ for the mass fractal dimension and $d_{w}=3.1$ for the anomalous diffusion exponent of CCA-clusters [2]. Note that the effective filler volume fraction $\Phi_{\mathrm{eff}}>\Phi \;$ is used in Equations (1) and (5), which considers the effective volume of the rigid phase of structured filler particles, e.g., carbon black or silica, according to the “occluded rubber concept” of Medalia [32]. Occluded rubber is defined as the rubber part of the rubber matrix that penetrates into the voids of the particles, which partially shields it from deformation. The second addend of Equation (5) takes into account that also fully broken clusters contribute to the strain amplification factor by the remaining particles. $\varphi \left(\xi_{\mu }\right)$ is the normalized cluster size distribution:

(6) $$\varphi \left(\xi_{\mu }\right)=4d\frac{\xi_{\mu }}{\langle \xi_{\mu }\rangle {}^{2}}\exp\left(-2\frac{\xi_{\mu }}{\langle \xi_{\mu }\rangle }\right)$$

This is a peaked cluster size distribution with $\langle \xi_{\mu }\rangle$ being the ensemble average in spatial direction μ. It is motivated by analytical results referring to Smoluchowski’s equation for the kinetics of cluster–cluster aggregation of colloids [33,34,35] (comp. also [7]). In the undeformed state it is assumed to be isotropic, i.e., $\varphi \left(\xi_{1}\right)=\varphi \left(\xi_{2}\right)=\varphi \left(\xi_{3}\right)\equiv \varphi \left(\xi \right)$.

**Figure 1:**
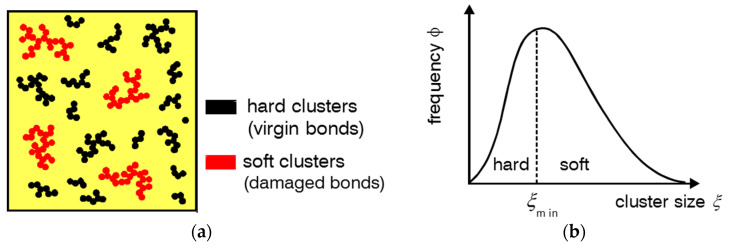
(**a**) Schematic view of the decomposition of filler clusters in hard and soft units for preconditioned samples and (**b**) cluster size distribution with the pre-strain dependent boundary size $x_{\min}$ between hard and soft clusters.

The model of stress softening and hysteresis assumes that the breakdown of filler clusters during the first deformation of the virgin samples is reversible, though the initial virgin state of filler–filler bonds is not recovered. This implies that, on the one side, the fraction of hard (virgin) filler clusters decreases with increasing pre-strain, leading to pronounced stress softening after the first deformation cycle. On the other side, the fraction of soft (reaggregated) filler clusters increases with rising pre-strain, which affects the filler-induced hysteresis. A schematic view of the decomposition of filler clusters in hard and soft units for preconditioned samples is shown in Figure 1.

The second addend of Equation (1) describes the filler-induced hysteresis. It considers the energy stored in the substantially strained soft filler clusters, which break under stress and reaggregate on retraction

(7) $$W_{A}\left(\varepsilon_{\mu }\right)=\overset{\partial \varepsilon /\partial t>0}{\underset{\mu }{\sum }}\frac{1}{2d}\int_{\xi_{\mu },\min}^{\xi_{\mu }\left(\varepsilon_{\mu }\right)}G_{A}\left(\xi_{\mu }^{’}\right)\varepsilon_{A,\mu }^{2}\left(\xi_{\mu }^{’},\varepsilon_{\mu }\right)\varphi \left(\xi_{\mu }^{’}\right)\;d\xi_{\mu }^{’}$$

$G_{A}$ is the elastic modulus and $\varepsilon_{A,\mu }$ is the strain of the fragile filler clusters in spatial direction μ. These quantities and their dependence on cluster size $x_{\mu }$ and external strain $\varepsilon_{\mu }$ will be specified in the next sections. In addition. The integral boundaries of Equations (5) and (7) have to be described more closely, which requires the consideration of elasticity and fracture of filler clusters in stretched elastomers.

### 2.2. Elasticity and Fracture of Filler Clusters in Stretched Elastomers

**Figure 2:**
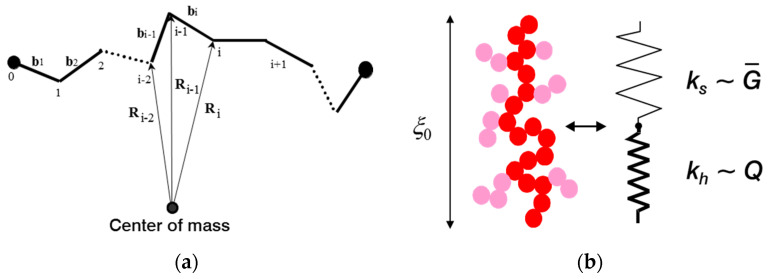
Schematic view of the Kantor–Webman model of flexible chains of arbitrary connected filler particles (**a**) and mechanical equivalence between a filler cluster and a series of soft and stiff molecular springs (**b**). The two springs with force constants $k_{s}$ and $k_{h}$ are related to bending–twisting- and tension deformations of filler–filler bonds referring to elastic constants $\bar{G}$ and Q, respectively.

For consideration of filler network breakdown in stretched rubbers, the elasticity and failure properties of tender filler clusters have to be evaluated in dependence of cluster size. This will be obtained by referring to the two-dimensional Kantor–Webman model of flexible chains of arbitrary connected filler particles [11] as represented in Figure 2a. We apply here a simplified generalization of this model to three dimensions, where on-plane bending, and off-plain twisting deformations of bonds are considered by a single bending–twisting term [20]. By identifying the three-dimensional flexible chain with the backbone of a CCA-cluster, the model can be applied for modeling the small-strain modulus of fractal filler networks, consisting of a space-filling configuration of CCA-clusters [7,8,9,10]. Here, we use it for a micromechanical description of CCA-clusters that are deformed in the stress field of a strained rubber matrix. Note that this is possible because the CCA-cluster backbone is not branched on large length scales, which is a typical result of cluster–cluster aggregation.

In our model two kinds of deformations of filler–filler bonds are considered, bending–twisting- and tension deformations. This corresponds to a mechanical equivalence between a filler cluster and a series of two molecular springs depicted schematically in Figure 2b. We will see that the bending–twisting deformation governs the elasticity while the tension deformation is sensitive for fracture. The total force constant of a cluster of size ξ_0_ with *N_B_* particles in the backbone reads:

(8) $$k_{\xi }={\left(\frac{1}{k_{h}}+\frac{1}{k_{s}}\right)}^{-1}$$

with the tension part given by:

(9) $$k_{h}=\frac{Q}{\sum_{i=1}^{N_{B}}{\left(\frac{\vec{F}}{F}\cdot {\vec{b}}_{i}\right)}^{2}\;}=\frac{Q}{N_{B}d^{2}\oint {\left(\frac{\vec{F}}{F}\cdot \frac{\vec{b}}{d}\right)}^{2}dS}=\frac{Q}{d^{2}g}{\left(\frac{d}{\xi_{0}}\right)}^{d_{f,B}}$$

The bending–twisting part reads:

(10) $$k_{s}=\frac{\bar{G}}{\sum_{i=1}^{N_{B}}{\left[\left(\frac{\vec{F}}{F}\times \vec{z}\right)\left({\vec{R}}_{i-1}-{\vec{R}}_{N_{B}}\right)\right]}^{2}\;}=\frac{\bar{G}}{N_{B}S_{⊥}^{2}}=\frac{\bar{G}}{d^{2}g’}{\left(\frac{d}{\xi_{0}}\right)}^{2+d_{f,B}}$$

Here, d is the bond length (particle size), $\vec{F}$ is the force and $g\equiv \oint {\left(\frac{\vec{F}}{F}\cdot \frac{\vec{b}}{d}\right)}^{2}dS$ is the average projection of bond vectors ${\vec{b}}_{i}$ on the direction of the force (0<g<1). $S_{⊥}^{2}\equiv \frac{1}{N_{B}}\overset{N_{B}}{\underset{i=1}{\sum }}{\left[\left(\frac{\vec{F}}{F}\times \vec{z}\right)\left({\vec{R}}_{i-1}-{\vec{R}}_{N_{B}}\right)\right]}^{2}\;$ is the average squared radius of gyration in direction perpendicular to the force and includes a unit vector $\vec{z}$ pointing perpendicular to the connecting vector $\left({\vec{R}}_{i-1}-{\vec{R}}_{N_{B}}\right)$. It scales with the squared cluster size, $S_{⊥}^{2}=g^{\prime }\xi_{0}^{2}$, with a scaling factor $0<g^{\prime }<1$. $\bar{G}$ and Q are elastic constants due to bending–twisting—and tension deformations, respectively. For the particle number the scaling relation $N_{B}={\left(\xi_{0}/d\right)}^{d_{f,B}}$ was used with $d_{f,B}=1.3$ being the backbone fractal dimension of CCA-clusters.

By comparing the exponents of Equations (9) and (10) one finds that the force constant $k_{s}$ decreases much more rapidly with cluster size $\xi_{0}$ than the force constants $k_{h}$. Accordingly, Equation (8) implies $k_{\xi }\approx k_{s}$ for sufficient large clusters, i.e., the stiffness of the cluster is determined by the bending–twisting deformations of bonds. This determines the following scaling law for the elastic modulus entering Equation (7):

(11) $$G_{A}\left(\xi_{0}\right)\approx \xi_{0}^{-1}k_{s}=\frac{\bar{G}}{d^{3}g^{\prime }}{\left(\frac{d}{\xi_{0}}\right)}^{3+d_{f,B}}≃\frac{\bar{G}}{d^{3}}{\left(\frac{d}{\xi_{0}}\right)}^{3+d_{f,B}}$$

This approximation without the tension term can be applied for sufficient large clusters with ${\left(\xi_{0}/d\right)}^{2+d_{f,B}}\gg {\left(\xi_{0}/d\right)}^{d_{f,B}}$. For the evaluation of the scaling factor $g^{\prime }$ in Equation (11) we have to consider the ensemble average of clusters. However, this will not be considered here, because we are mainly interested in the scaling exponents.

The stretching of the clusters can be evaluated in the same approximation [7]:

(12) $$\Delta l_{\xi }=\Delta l_{s}+\Delta l_{h}=\Delta l_{b}\left(\frac{k_{b}}{k_{s}\left(\xi_{0}\right)}+gN_{B}\right)\approx \Delta l_{b}\frac{g^{\prime }Q}{\bar{G}}{\left(\frac{\xi_{0}}{d}\right)}^{2+d_{f,B}}$$

Here, we have introduced the stretching $\Delta l_{b}$ and force constant $k_{b}\equiv Q/d^{2}$ related to stretching of the single bonds. In addition. we used the equilibrium conditions for the force $\Delta l_{s}/\Delta l_{b}=k_{b}/k_{s}\left(\xi_{0}\right)\;$ and $\Delta l_{h}/\Delta l_{b}=k_{b}/k_{h}\left(\xi_{0}\right)=gN_{B}$. In the next section we will use Equation (12) for describing the fracture of filler clusters by relating it to the fracture of bonds under tension.

**Figure 3:**
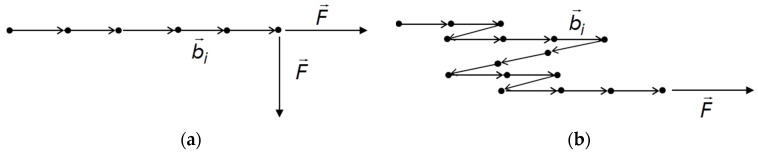
Illustration of the cluster mechanics by simple examples: (**a**) Linear chain and (**b**) one dimensional random walk.

Examples:

The cluster mechanics described by Equations (8)–(10) shall be illustrated by two simple examples which are depicted in Figure 3. In Figure 3a a linear chain is considered with the force $\vec{F}$ pointing perpendicular or alternatively in direction of the chain. In the first case we have g=0 and $\vec{F}\times \vec{z}$ points into the direction of the chain. This implies for the force constant and the deformation:

(13) $$k_{\xi }=k_{s}=12\frac{\bar{G}}{N_{B}{\left(N_{B}d\right)}^{2}}\;\to \;\Delta {\vec{l}}_{s}=\frac{\vec{F}}{k_{s}}=\frac{\vec{F}}{12d\bar{G}}{\left(N_{B}d\right)}^{3}$$

With $g^{\prime }=1/12$ being the ratio between the squared radius of gyration and the squared length $L^{2}={\left(N_{B}d\right)}^{2}$ of a linear chain. Accordingly, the force constants $k_{s}$ drops with the 3rd power of the length.

In the second case, where $\vec{F}$ points parallel to the chain, we have g=1 and $\vec{F}\times \vec{z}$ points perpendicular to the chain. This implies ${\bar{S}}_{⊥}=0$ yielding:

(14) $$k_{\xi }=k_{h}=\frac{Q}{N_{B}d^{2}}\;\to \;\Delta {\vec{l}}_{h}=\frac{\vec{F}}{k_{h}}=N_{B}\frac{d^{2}\vec{F}}{Q}$$

As expected, the deformation increases linear with the number of bonds.

In Figure 3b we consider the case where a random walk structure of the chain is realized, corresponding to three 1-dimensional random walks with $N_{B}/3$ particles. The average projection g is then given by $g=\oint {\left(\frac{\vec{F}}{F}\cdot \frac{\vec{b}}{d}\right)}^{2}dS$ = 1/3. The ratio between the ensemble average of the squared radius of gyration $\langle R_{g}^{2}\rangle$ and the squared end-to-end distance $R^{2}=N_{B}d^{2}$ is evaluated as $g^{\prime }=1/6$ (see e.g., Chapter 2 in [36]). This implies for the force constant:

(15) $$k_{\xi }={\left(\frac{N_{B}^{2}d^{2}}{6\bar{G}}+\frac{N_{B}d^{2}}{3Q}\right)}^{-1}$$

For the deformation under tension of the 1-dimensional random walk shown in Figure 3b one obtains:

(16) $$\Delta {\vec{l}}_{h}=\frac{\vec{F}}{k_{h}}=\frac{N_{B}}{3}\frac{d^{2}\vec{F}}{Q}$$

For the more general case that the force points in arbitrary direction also the bending–twisting deformations of bonds must be taken into account by referring to the full force constant $k_{\xi }$ of Equation (15).

### 2.3. Evaluation of Boundary Cluster Size and Cluster Stress

In view of introducing a fracture criterion for strained clusters, we assume that the tension of bonds is a much more critical deformation compared to bending and twisting, since it separates the filler particles from each other. Equation (12) relates the total stretching of a cluster to the stretching of the bonds and can therefore be used for evaluation of the failure strain of the cluster by defining a fracture criterion for the bonds. We will introduce here two different fracture criteria, which will be denoted “monodisperse” and “hierarchical”.

In the first approach all bonds are considered to be equal (monodisperse) having the same strength. Then, the failure strain $\varepsilon_{f,b}$of the bonds is given by the critical stretch of the bonds $\Delta l_{f,b}$ in relation to the bond length: $\varepsilon_{f,b}^{m}\equiv \Delta l_{f,b}/d$. This implies for the failure strain of the cluster:

(17a) $$\varepsilon_{f,\xi }\equiv \frac{\Delta l_{f,\xi }}{\xi_{0}}\approx \varepsilon_{f,b}^{m}\frac{g^{\prime }Q}{\bar{G}}{\left(\frac{\xi_{0}}{d}\right)}^{1+d_{f,B}}$$

In contrast, the hierarchical model takes into account that a hierarchy of bond strengths develops during cluster–cluster aggregation, because the mobility of the clusters decreases with cluster size. Accordingly, the first bond formed between two particles is the strongest while successive bonds formed between the growing sub-clusters become weaker and weaker. The last bond formed in the cluster is the weakest and will break first under tension. This effect is taken into account by the hierarchical fracture criterion, where the failure strain $\varepsilon_{f,b}$ of the bonds is defined in relation to the cluster size, which is the only relevant length scale in our model: $\varepsilon_{f,b}^{h}\equiv \Delta l_{f,b}/\xi_{0}$. This implies that the failure strain of the cluster increases more rapidly with cluster size compared to the monodisperse case:

(17b) $$\varepsilon_{f,\xi }\equiv \frac{\Delta l_{f,\xi }}{\xi_{0}}\approx \varepsilon_{f,b}^{h}\frac{g^{\prime }Q}{\bar{G}}{\left(\frac{\xi_{0}}{d}\right)}^{2+d_{f,B}}$$

For the evaluation of the boundary cluster size between broken and unbroken clusters in stretched rubbers, we assume that a stress equilibrium is realized between the strain amplified rubber matrix and the clusters $\sigma_{R,\mu }\left(\varepsilon_{\mu }\right)=G_{A}\varepsilon_{A,\mu }\left(\varepsilon_{\mu }\right)$. With the scaling relation Equation (11) for the elastic modulus of the clusters this delivers for the cluster strain:

(18) $$\varepsilon_{A,\mu }\left(\varepsilon_{\mu }\right)=G_{A}^{-1}{\hat{\sigma }}_{R,\mu }\left(\varepsilon_{\mu }\right)\approx \frac{g^{\prime }d^{3}}{\bar{G}}{\left(\frac{\xi_{0}}{d}\right)}^{3+d_{f,B}}{\hat{\sigma }}_{R,\mu }\left(\varepsilon_{\mu }\right)$$

Here we have replaced the rubber stress by a relative stress with respect to the minimum strain:

(19) $${\hat{\sigma }}_{R,\mu }\left(\varepsilon_{\mu }\right)\equiv \left(\sigma_{R,\mu }\left(\varepsilon_{\mu }\right)-\sigma_{R,\mu }\left(\varepsilon_{\mu ,\min}\right)\right)$$

This ensures that the stretching of clusters in spatial direction μ starts at the minimum strain $\varepsilon_{\mu ,\min}$ for each cycle. Here, we assume that clusters reaggregate into a stress-free state at minimum strain.

A comparison of the exponents in Equations (18) and (17) makes clear that the strain of the clusters under external strain increases faster with cluster size than the failure strain, in both cases. This implies that large clusters break first followed by smaller ones, i.e., the boundary cluster size between broken and unbroken clusters $\xi_{\mu }\left(\varepsilon_{\mu }\right)$ moves from larger to smaller values with increasing strain. It is obtained by equating the cluster strain to the failure strain. This yields for the two fracture criteria:

(20a) $${\left(\frac{\xi_{\mu }\left(\varepsilon_{\mu }\right)}{d}\right)}^{2}=\frac{Q\varepsilon_{f,b}^{m}}{d^{3}{\hat{\sigma }}_{R,\;\mu }\left(\varepsilon_{\mu }\right)}\equiv \frac{s_{d}}{{\hat{\sigma }}_{R,\;\mu }\left(\varepsilon_{\mu }\right)}$$

And

(20b) $$\frac{\xi_{\mu }\left(\varepsilon_{\mu }\right)}{d}=\frac{Q\varepsilon_{f,b}^{h}}{d^{3}{\hat{\sigma }}_{R,\;\mu }\left(\varepsilon_{\mu }\right)}\equiv \frac{s_{d}}{{\hat{\sigma }}_{R,\;\mu }\left(\varepsilon_{\mu }\right)}$$

Here, $s_{d}$ is defines as the fracture stress under tension of bonds, i.e., the tensile strength of damaged filler–filler bonds. The boundary cluster size $\xi_{\mu }\left(\varepsilon_{\mu }\right)$ applies for the integral boundaries of Equation (7), describing the filler-induced hysteresis due to the successive breakdown of soft filler clusters with damaged filler–filler bonds.

Similar expressions are found for the upper boundary of Equation (5), but now the tensile strength $s_{v}$ of virgin filler–filler bonds is entering:

(21a) $${\left(\frac{\xi_{\mu ,\min}}{d}\right)}^{2}=\frac{\tilde{Q}{\tilde{\varepsilon }}_{f,b}^{m}}{d^{3}{\hat{\sigma }}_{R,\;\mu }\left(\varepsilon_{\mu ,\max}\right)}\equiv \frac{s_{v}}{{\hat{\sigma }}_{R,\;\mu }\left(\varepsilon_{\mu ,\max}\right)}$$

And

(21b) $$\frac{\xi_{\mu ,\min}}{d}=\frac{\tilde{Q}{\tilde{\varepsilon }}_{f,b}^{h}}{d^{3}{\hat{\sigma }}_{R,\;\mu }\left(\varepsilon_{\mu ,\max}\right)}\equiv \frac{s_{v}}{{\hat{\sigma }}_{R,\;\mu }\left(\varepsilon_{\mu ,\max}\right)}$$

The elastic constant and failure strains are denoted by $\tilde{Q}$ and ${\tilde{\varepsilon }}_{f,b}$, respectively. Note that the tensile strength of virgin filler–filler bonds must be larger than the tensile strength of damaged bonds, i.e., $s_{v}>s_{d}$. Equation (21a) or (21b) together with Equation (5) define the amplification of the rubber matrix, and thus stress—softening effects of the model. Solving this set of equations for *σ* requires iterative methods, e.g., Newton iteration.

By referring to the stress equilibrium between the strain amplified rubber matrix and the clusters, ${\hat{\sigma }}_{R,\mu }\left(\varepsilon_{\mu }\right)=G_{A}\varepsilon_{A,\mu }\left(\varepsilon_{\mu }\right)$, the cluster stress $\sigma_{A,\mu }$ responsible for filler-induced hysteresis is obtained by differentiation of Equation (7) with respect to cluster strain:

(22) $$\begin{array}{l} \sigma_{A,\nu }\equiv \frac{\partial W_{A}}{\partial \varepsilon_{A,\nu }}=\overset{\frac{\partial \varepsilon }{\partial t}>0}{\underset{\mu }{\sum }}\frac{1}{d}\int_{d{\left(\frac{s_{d}}{{\hat{\sigma }}_{R,\mu }\left(\varepsilon_{\mu ,\max}\right)}\right)}^{\alpha }}^{d{\left(\frac{s_{d}}{{\hat{\sigma }}_{R,\mu }\left(\varepsilon_{\mu }\right)}\right)}^{\alpha }}G_{A}\left(\xi_{\mu }\right)\varepsilon_{A,\mu }\left(\xi_{\mu },\varepsilon_{\mu }\right)\frac{\partial \varepsilon_{A,\mu }\left(\xi_{\mu }\right)}{\partial \varepsilon_{A,\nu }\left(\xi_{\mu }\right)}\varphi \left(\xi_{\mu }\right)\;d\xi_{\mu }=\\ \overset{\frac{\partial \varepsilon }{\partial t}>0}{\underset{\mu }{\sum }}{\hat{\sigma }}_{R,\mu }\left(\varepsilon_{\mu }\right)\langle \frac{\partial \varepsilon_{A,\mu }}{\partial \varepsilon_{A,\nu }}\rangle \frac{1}{d}\int_{d{\left(\frac{s_{d}}{{\hat{\sigma }}_{R,\mu }\left(\varepsilon_{\mu ,\max}\right)}\right)}^{\alpha }}^{d{\left(\frac{s_{d}}{{\hat{\sigma }}_{R,\mu }\left(\varepsilon_{\mu }\right)}\right)}^{\alpha }}\varphi \left(\xi_{\mu }\right)\;d\xi_{\mu }\; \end{array}$$

The exponent α takes the two fracture criteria into account, i.e., α=1/2 for the “monodisperse” model and α=1 for the “hierarchical” model. In addition. we assume that the clusters, on average, deform like the sample:

(23) $$\langle \frac{\partial \varepsilon_{A,\mu }}{\partial \varepsilon_{A,\nu }}\rangle =\frac{\partial \varepsilon_{\mu }}{\partial \varepsilon_{\nu }}$$

The sum in Equation (22) runs over stretching directions, only, implying that the up- and down cycles are different. The cluster stress of the upcycle is positive while the downcycle gives a negative contribution, producing the filler-induced hysteresis.

For uniaxial deformations, realized on microscales $\lambda_{2}=\lambda_{3}=\lambda_{1}^{-1/2}$; $\partial \lambda_{2}=\partial \lambda_{3}=-1/2\;\lambda_{1}^{-3/2}\partial \lambda_{1}$) and macroscales $1+\varepsilon_{2}=1+\varepsilon_{3}={\left(1+\varepsilon_{1}\right)}^{-1/2}$; $\partial \varepsilon_{2}=\partial \varepsilon_{3}=-1/2\;{\left(1+\varepsilon_{1}\right)}^{-3/2}\partial \varepsilon_{1}$, the cluster stress in stretching direction for the upcycle ($\partial \varepsilon_{1}/\partial t>0$) is obtained as:

(24) $$\sigma_{A,1}^{\mathrm{up}}={\hat{\sigma }}_{R,1}\left(\varepsilon_{1}\right)\frac{1}{d}\int_{d{\left(\frac{s_{d}}{{\hat{\sigma }}_{R,1}\left(\varepsilon_{1,\max}\right)}\right)}^{\alpha }}^{d{\left(\frac{s_{d}}{{\hat{\sigma }}_{R,1}\left(\varepsilon_{1}\right)}\right)}^{\alpha }}\varphi \left(\xi_{1}\right)\;d\xi_{1}\;$$

For the down cycle, the lateral directions contribute to the cluster stress ($\partial \varepsilon_{2}$/∂*t* > 0; $\partial \varepsilon_{3}$/∂*t* > 0):

(25) $$\sigma_{A,1}^{\mathrm{down}}=2{\hat{\sigma }}_{R,2}\left(\varepsilon_{1}\right)\left(-\frac{1}{2}{\left(1+\varepsilon_{1}\right)}^{-\frac{3}{2}}\right)\frac{1}{d}\int_{d{\left(\frac{s_{d}}{{\hat{\sigma }}_{R,2}\left(\varepsilon_{1,\max}\right)}\right)}^{\alpha }}^{d{\left(\frac{s_{d}}{{\hat{\sigma }}_{R,2}\left(\varepsilon_{1}\right)}\right)}^{\alpha }}\varphi \left(\xi_{2}\right)\;d\xi_{2}\;$$

With $\varphi \left(\xi_{1}\right)=\varphi \left(\xi_{2}\right)=\varphi \left(\xi_{3}\right)\equiv \varphi \left(\xi \right)$. This gives a negative stress contribution, which must be subtracted from the rubber stress. It can also be expressed by the rubber stress $\sigma_{R,1}$ in stretching direction. By assuming that the same energy is needed for stretching in 1-direction and compressing in 2- and 3-direction to obtain a final deformed state, the following relation is derived:

(26) $${\hat{\sigma }}_{R,2}\left(\varepsilon_{2}\right)\equiv \sigma_{R,2}\left(\varepsilon_{2}\right)-\sigma_{R,2}\left(\varepsilon_{2,\min}\right)=-\lambda_{1}^{3/2}\sigma_{R,1}\left(\varepsilon_{1}\right)-\lambda_{1,\max}^{3/2}\sigma_{R,1}\left(\varepsilon_{1,\max}\right)$$

Finally, for the evaluation of the (measured) total stress we have to consider an additional set stress $\sigma_{\mathrm{set}}$ that appears as a remaining stress in the undeformed state after stretching and retraction. Note that this is also found for unfilled rubbers and depends on temperature and stretching rate. It probably results from long time relaxation effects of the polymer network. We introduce it in a purely empirical manner for the case of uniaxial deformations:

(27) $$\sigma_{\mathrm{set},1}=s_{\mathrm{set},0}\left(\sqrt{\varepsilon_{1,\max}}-\sqrt{\varepsilon_{1,\min}}\right)$$

Then for uniaxial deformations the total stress reads:

(28) $$\sigma_{\mathrm{tot},1}\left(\varepsilon_{1}\right)=\sigma_{R,1}\left(\varepsilon_{1}\right)+\sigma_{A,1}^{\mathrm{up}/\mathrm{down}\;}\left(\varepsilon_{1}\right)+\sigma_{\mathrm{set},1}\left(\varepsilon_{1,\max},\varepsilon_{1,\min}\right)$$

The stress of the rubber matrix $\sigma_{R,1}$ is given by Equation (4) with $\lambda_{\mu }=1+X\left(\varepsilon_{\mu ,\max}\right)\varepsilon_{\mu }$ and strain amplification factor $X\left(\varepsilon_{\mu ,\max}\right)$ specified by Equation (5). The cluster stress $\sigma_{A,1}$ depends on the direction of straining and is determined by Equations (24) and (25) for up and down, respectively. The theory presented here describes the complex quasi-static deformation behavior of filler reinforced elastomers for repeated stretching with increasing amplitude. A more general formulation of the DFM that applies for arbitrary deformation histories requires an additional term for the free energy density of soft filler clusters Equation (7), which considers the relaxation of cluster stress upon retraction [15,16]. For a test of the theory the present formulation for repeated stretching with increasing amplitude is sufficient because all open parameters are entering already and the extension to arbitrary deformation histories requires no additional fitting parameters.

### 2.4. Frame-Independent Formulation of Stress-Softening for Fast FEM Simulations

The implementation of the DFM into a FEM algorithm faces several problems. First, the DFM is a microscopic theory, where stresses are calculated by differentiation of the free energy density with respect to the internal strain variables $\lambda_{\mu }$ and $\lambda_{A,\mu }$, respectively. This is in discrepancy to continuum mechanical considerations, where corresponding differentiations have to be performed with respect to external strain variables. Second, the DFM is formulated in the main axis system and requires stress contributions of different directions, especially for description of filler-induced hysteresis, which all sum up to produce close cycles. This can hardly be transferred to a pure tensorial formulation. Nevertheless, a FEM implementation of the DFM was obtained by referring to the concept of representative directions, which considers uniaxial deformations along fibers in different spatial directions [24,25]. However, the computational cost of this workaround is very large, and the efficiency is low. Therefore, we want to focus here on a frame-independent tensor formulation of the stress-softening part of the DFM for fast and efficient FEM simulations.

In a first step we put the strain amplification factor $X_{\max}\equiv X\left(\varepsilon_{\mu ,\max}\right)$ in front of the deformation invariants, appearing in the free energy density of the extended non-affine tube model Equation (2) and replace the internal strain $\lambda_{\mu }=1+X\left(\varepsilon_{\mu ,\max}\right)\varepsilon_{\mu }$ by the external strain $\lambda_{\mu }=1+\varepsilon_{\mu }$. This follows the ideas of Einstein [37] and Domurath et al. [38] and is done in close correlation to the evaluations in [22]. The free energy density reads:

(29) $$W_{R}=\frac{G_{c}}{2}\left\{\frac{X_{\max}{\bar{I}}_{1}\left(1-\frac{T_{e}}{n_{e}}\right)}{1-\frac{T_{e}}{n_{e}}X_{\max}{\bar{I}}_{1}}+\ln\left[1-\frac{T_{e}}{n_{e}}X_{\max}{\bar{I}}_{1}\right]\right\}+2G_{e}X_{\max}{\bar{I}}^{*}$$

with the (frame-independent) first invariant of the left Cauchy–Green tensor:

(30) $${\bar{I}}_{1}\equiv \lambda_{1}^{2}+\lambda_{2}^{2}+\lambda_{3}^{2}-3$$

and the (frame-independent) generalized invariant:

(31) $${\bar{I}}^{*}\equiv \lambda_{1}^{-1}+\lambda_{2}^{-1}+\lambda_{3}^{-3}-3$$

Here, $\lambda_{\mu }=1+\varepsilon_{\mu }$ is the external strain of the sample. A frame-independent formulation of the strain amplification factor $X_{\max}$ is obtained similar to Equation (5) by replacing the relative stress ${\hat{\sigma }}_{R,\mu }\left(\varepsilon_{\mu ,\max}\right)$ used for the calculation of the boundary cluster size $x_{\mu ,\;\min}$ by the Frobenius norm $∥\sigma_{R}\left(\varepsilon_{\max}\right)∥$ of the engineering stress.

(32) $$X\left(\varepsilon_{\max}\right)=1+c\Phi_{\mathrm{eff}}^{\frac{2}{3-d_{f}}}\frac{1}{d}\left[\int_{0}^{d{\left(s_{v}/\sigma_{R}\left(\varepsilon_{\max}\right)\right)}^{\alpha }\;}{\left(\frac{\xi }{d}\right)}^{d_{w}-d_{f}}\varphi \left(\xi \right)d\xi +\int_{d{\left(s_{v}/\sigma_{R}\left(\varepsilon_{\max}\right)\right)}^{\alpha }}^{\infty }\varphi \left(\xi \right)d\xi \right]$$

The iteration procedure for the evaluation of stresses (compare Equation (21a) or (21b) together with Equation (5)) is then replaced by its tensorial analog:

(33) $$\sigma_{R,\mu }\left(\varepsilon_{\mu ,\max}\right)=f\left(X_{\max}\left(\sigma_{R,\mu }\left(\varepsilon_{\mu ,\max}\right)\right)\right)\;\to \;\sigma_{R}\left(\varepsilon_{\max}\right)=f\left(X_{\max}\left(\sigma_{R}\left(\varepsilon_{\max}\right)\right)\right)$$

The free energy density Equation (29) can be used in a standard continuum mechanical sense for the evaluation of stresses and tangent vectors. It can be further simplified by omitting the logarithmic term, which gives a minor contribution to the stress upturn. In addition. The generalized invariant can be approximated by the square root of the second invariant, which avoids the calculation of eigenvalues [39].

## 3. Experimental and Fitting Procedure

### 3.1. Materials

EPDM/A (“aging”) samples were prepared by using an amorphous-type ethylene–propylene-diene rubber (EPDM, Keltan 4450) filled with 50-phr (parts per hundred mass parts rubber) carbon black (N339). The rubber has a Mooney viscosity of 46 MU (at 125 °C) and consists of 52% ethylene and 4.3% ethylidene norbornene. Moreover, 3 phr zinc oxide, 1 phr stearic acid and 1.5 phr *N*-isopropyl-*N*’-phenyl-1,4-phenylenediamine (IPPD, aging protection) were added. The curing system consists of 1.8-PHR sulfur, 1.5 phr 1,3-diphenylguanidine (DPG) and 2.4 phr *N*-cyclohexyl-2-benzothiazolylsulfenamide (CBS, accelerator). EPDM/CB (“carbon black”) consists of the same polymer, additives and curing system, but has varying amounts of carbon black (N339): 20, 40 and 60 phr.

### 3.2. Mixing and Sample Preparation

All ingredients except of the vulcanization system were mixed in an internal mixer of type GK 1,5E (Werner & Pfleiderer Gummitechnik GmbH, Freudenberg, Germany) at a loading of 75% and a temperature of less than 140 °C. The vulcanization system was added in a second step on a 150*350RR roller mill (KraussMaffei Berstorff GmbH, Hannover, Germany).

All samples were cured in a heated press of type WLP 63/3,5/3 (Wickert Maschinenbau GmbH, Landau, Germany) at 160 °C up to the t_90%_ time, where 90% of the torque obtained from a vulcameter measurement is reached (17:23 min). One minute curing time was added per millimeter sample thickness to account for heat diffusion.

From EPDM/A dumbbell test specimen were prepared. These were stored under thermo-oxidative aging conditions at 130 °C in an air-ventilated oven for 0, 1, 3, 7 and 14 days.

### 3.3. Test Methods and Fitting

Quasi-static multi-hysteresis experiments (multiple deformation cycles up to different strain levels) were performed in a Zwick 1445 (Zwick Roell, Ulm, Germany) universal testing machine using a crosshead speed of 20 mm/min. Every strain level was repeated five times. From EPDM/CB tensile test specimen of S2 type were prepared to achieve strains, which are not accessible using dumbbell samples. Multi-hysteresis experiments at 100 mm/min crosshead speed were performed in the same stretching machine. In both cases, the 5th cycles were separated for fittings with the DFM, which can be considered as equilibrium cycles.

The adaptation of stress-strain cycles to Equation (28) was performed by minimization of the error functional $\chi^{2}=\underset{\mathrm{cycles}}{\sum }\underset{n}{\sum }{\left(y_{\mathrm{mod},n}-y_{\exp,\;n\;}\right)}^{2}$, where $y_{\mathrm{mod},\;n}$ and $y_{\exp,\;n}$ represent the nth model and experimentally obtained 1. Piola–Kirchhoff (“engineering”) stress data point. The set stress parameter s_set,0_ was included in the fitting procedure as defined by referring to Equation (27). The remaining two stress contributions of Equation (28), which involve seven fitting parameters, were obtained iteratively by using Equations (4)–(6) for the intrinsic stress of the rubber matrix $\sigma_{R,1}$ and Equations (24) and (25) for the evaluation of cluster stress $\sigma_{A,1}$. Note that both stress contributions involve the same cluster size distribution, which stabilizes the fitting procedure, significantly. The front factor of Equation (5) was fixed as c=2.5 according to the Einstein equation [37] and the exponent was approximated as $d_{w}-d_{f}\approx 1$ to allow for an analytical solution of the integral. The three fitting parameters describing the rubber elastic network are the crosslink modulus $G_{c}$, the topological constraint modulus $G_{e}$ and the effective chain length for finite extensibility $n_{\mathrm{eff}}\equiv n_{e}/T_{e}$. The filler clusters are described by four fitting parameters, i.e., the effective filler volume fraction $\Phi_{\mathrm{eff}}$, the average cluster size $x_{0}\equiv \langle x_{\mu }\rangle /d$, the tensile strength of virgin filler–filler bonds $s_{v}$ and the tensile strength of damaged filler–filler bonds $s_{d}$.

## 4. Results and Discussion

### 4.1. Micromechanical Investigations of Thermo-Oxidative Aging

The thermo-oxidative aging of elastomers is of high technological interest for the rubber industry, because it mostly increases the hardness of the samples and has a negative effect on the fracture toughness or crack resistance. This limits the life time of rubber goods, significantly. The reason for this property losses are mainly seen in a change of the rubber–elastic network due to post-curing, but the often-accompanied increase in electrical conductivity indicates that also the carbon black network is altering during thermo-oxidative aging of elastomers. This is hardly to distinguish by standard measurement techniques since changes of the polymer- or filler network structure are difficult to detect. We therefore refer here to the evaluation of micromechanical material parameters, which are obtained by fitting the stress–strain response of the aged samples to the DFM.

**Figure 4:**
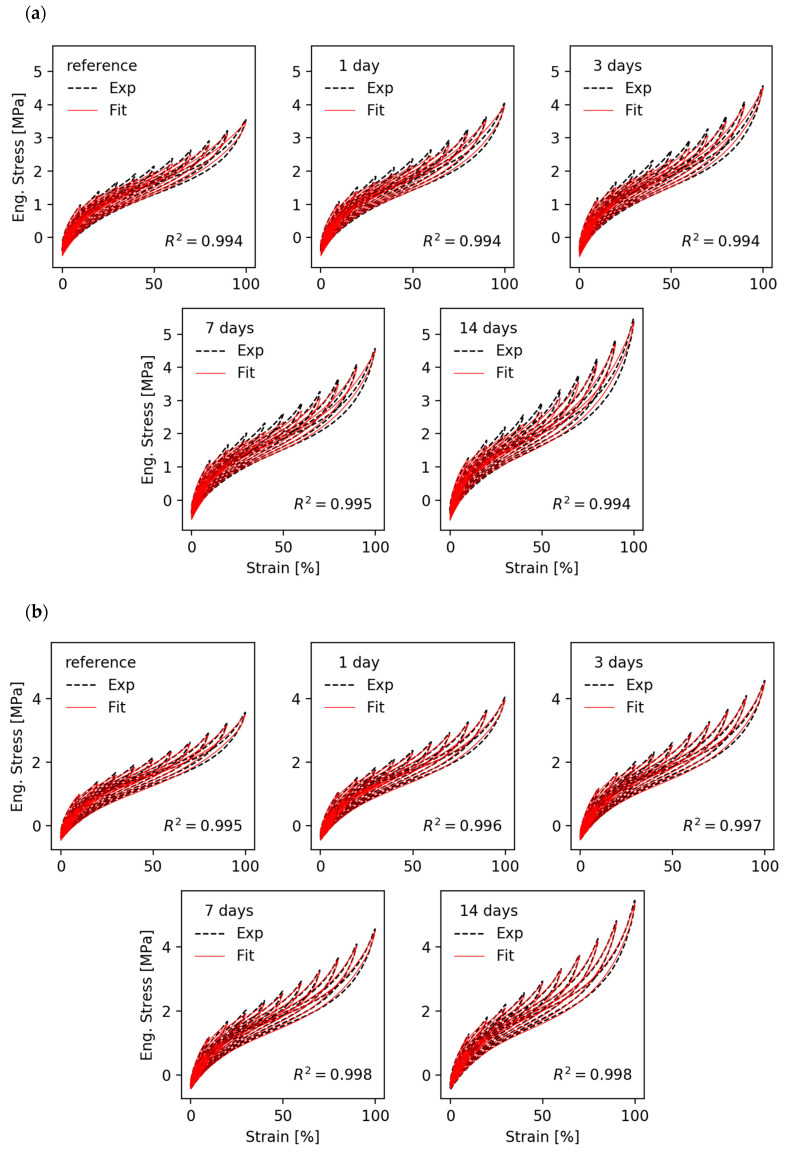
Fit of stress–strain cycles of ethylene–propylene-diene rubber EPDM/A samples for various aging times (**a**) with the “monodisperse” bond fracture model (α=1/2) and (**b**) with the “hierarchical” bond fracture model (α=1).

Figure 4 shows a series of fittings of multi-hysteresis stress–strain cycles of EPDM/A samples stored under thermo-oxidative aging conditions at 130 °C for 0, 1, 3, 7 and 14 days. In Figure 4a, the “monodisperse” bond fracture model (α=1/2) is used while Figure 4b refers to the “hierarchical” bond fracture model (α=1). Before we discuss the effect of thermomechanical aging on material parameters, we will first consider the quality of the fits for the two different bond fracture criteria. The “monodisperse” model depicted in Figure 4a delivers fair agreement between fits and experimental data with correlation coefficients between *R*^2^ = 0.994 and 0.995, but systematic deviations are seen, e.g., for the peak stresses in the medium strain regime. For the “hierarchical” model shown in Figure 4b, the fits are significantly better with correlation coefficients between *R*^2^ = 0.995 and *R*^2^ = 0.998. This indicates that the “hierarchical” model is more suited for describing the stress–strain cycles of filler reinforced elastomers. Even in the case of aged samples we get excellent adaptations in the small and medium strain regime up to 100% strain. Therefore, we will mainly focus on the “hierarchical” model with bond fracture exponent α=1 for the discussion of thermo-oxidative aging effects on a microscopic level. Nevertheless, we will see that the “monodisperse” model delivers similar values and trends of the fitting parameters.

**Figure 5:**
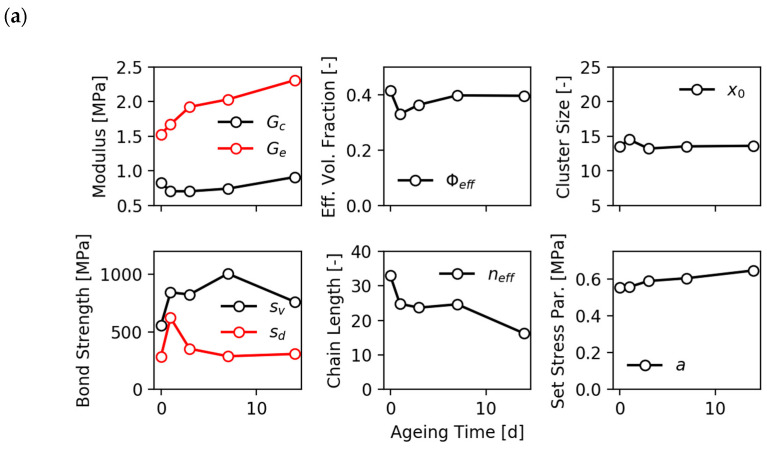

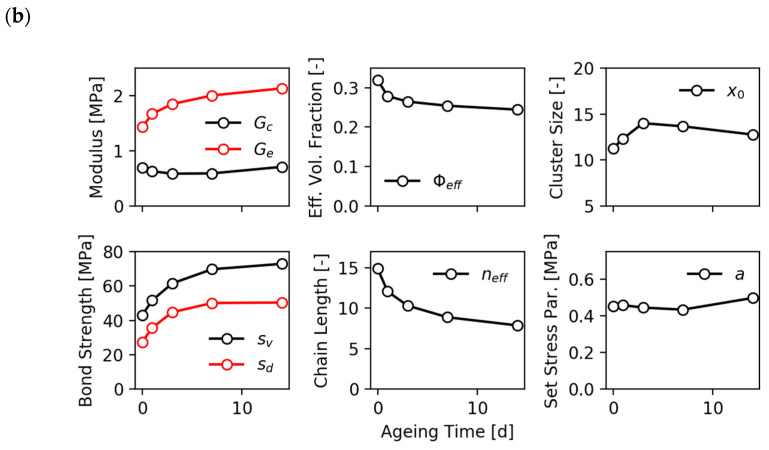
Evolution of fitting parameters obtained with (**a**) the “monodisperse” bond fracture model (α=1/2) and (**b**) the “hierarchical” bond fracture model (α=1) of the EPDM/A samples from Figure 4 for various aging times.

The stress–strain data in Figure 4 show that the average stress level increases with increasing aging time. The reason for this hardening of the samples shall be analyzed by referring to the evolution of fitting parameters that are depicted in Figure 5. The fitting parameters obtained with the “monodisperse” bond fracture model (α=1/2) are shown in Figure 5a and those from the “hierarchical” bond fracture model (α=1) in Figure 5b. Obviously, the “hierarchical” bond fracture model in Figure 5b delivers a smoother evolution of fitting parameters, which correlates with the higher correlation coefficients of the fits in Figure 4b. This confirms our view that the “hierarchical” bond fracture model is more suited for the discussion of aging effects. Looking first at the crosslink and topological constraint moduli of the polymer network, $G_{c}$ and $G_{e}$, we see that the former remains almost constant while the later increases successively with aging time. This indicates that the post-curing effect is not pronounced for the EPDM samples used in this study, but the topological constraints of the chains increase with aging time possibly due to an increasing number of entanglements close to the carbon black particles (surface-induced entanglements). This correlates with the observed decrease of the effective chain length, $n_{\mathrm{eff}}\equiv n_{e}/T_{e}$, since the segment number $n_{e}$ between successive entanglements decreases with aging time if $G_{e}$ increases ($G_{e}∼1/n_{e}$). However, it must be noted that it is difficult to distinguish between the two parameters $G_{c}$ and $G_{e}$ in the frame of the DFM, since both act in a similar way.

$n_{\mathrm{eff}}\equiv n_{e}/T_{e}$. The filler clusters are described by four fitting parameters, i.e., the effective filler volume fraction $\Phi_{\mathrm{eff}}$, the average cluster size $x_{0}\equiv \langle x_{\mu }\rangle /d$, the tensile strength of virgin filler–filler bonds $s_{v}$ and the tensile strength of damaged filler–filler bonds $s_{d}$.

An additional significant effect of thermo-oxidative aging is observed for the strength of virgin and damaged filler–filler bonds, $s_{v}$ and $s_{d}$, which both increase systematically with aging time. This is clearly seen in the case of the “hierarchical” bond fracture model (α=1). It indicates that a relaxation of bonds takes place during heating of the samples at 130 °C, leading to more stable filler–filler joints. Note that this is related to confined polymer between the filler particles, which is assumed to be in a glassy-like state at room temperature [7] but can relax at elevated temperature. This relaxation process has been investigated recently by online dielectric spectroscopy during heat treatment of carbon black filled EPDM [40]. The increase of $s_{v}$ and $s_{d}$ with aging time observed in Figure 5b results in stronger stress-softening and hysteresis effects of the aged samples. Two further parameters describing the aging of the filler network are the effective filler volume fraction $\Phi_{\mathrm{eff}}$ and the mean cluster size $\;x_{0}$. The former decreases slightly with aging time, but remains larger than the real filler volume fraction, as expected ($\Phi_{\mathrm{eff}}>\Phi \approx 0.2$). This indicates a slight decrease of the occluded rubber during aging, which is hidden in the voids of the filler particles and acts like additional filler. The mean cluster size lies in a reasonable range of about 10 to 15 particle diameters and goes through a weak maximum with increasing aging time. This can be related to flocculation effects and restructuring of the filler network

### 4.2. Frame-Independent Model of Stress Softening

For the discussion of the frame-independent model of stress softening we will focus on the “hierarchical” bond fracture model with α=1, only. For the analysis of the stress-softening effect, described here, we refer to the EPDM/CB samples with varying amount of carbon black. Note that the hysteresis is not included in this model.

**Figure 6:**
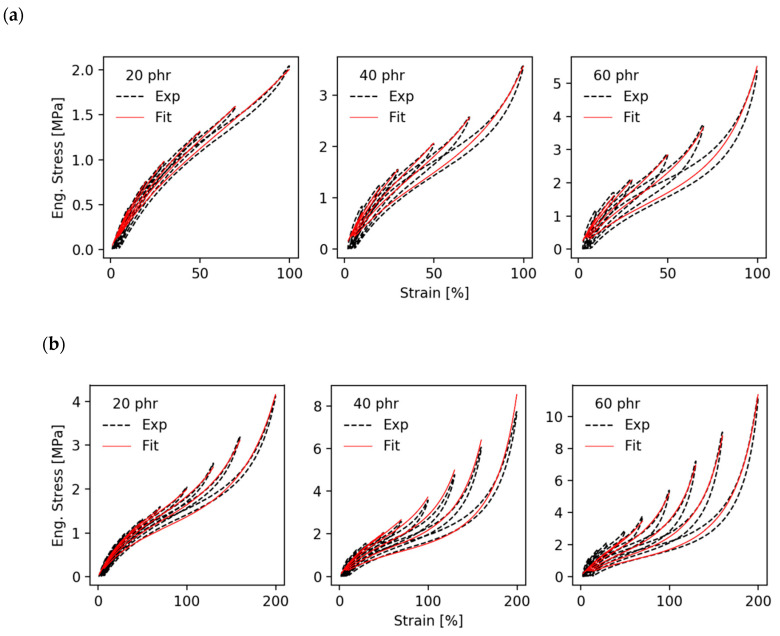
Fit of stress–strain cycles of EPDM/CB samples filled with various amounts of carbon black (N339) with the frame-independent model of stress softening (α=1) data up to 100% strain and (**b**) up to 200% strain.

**Figure 7:**
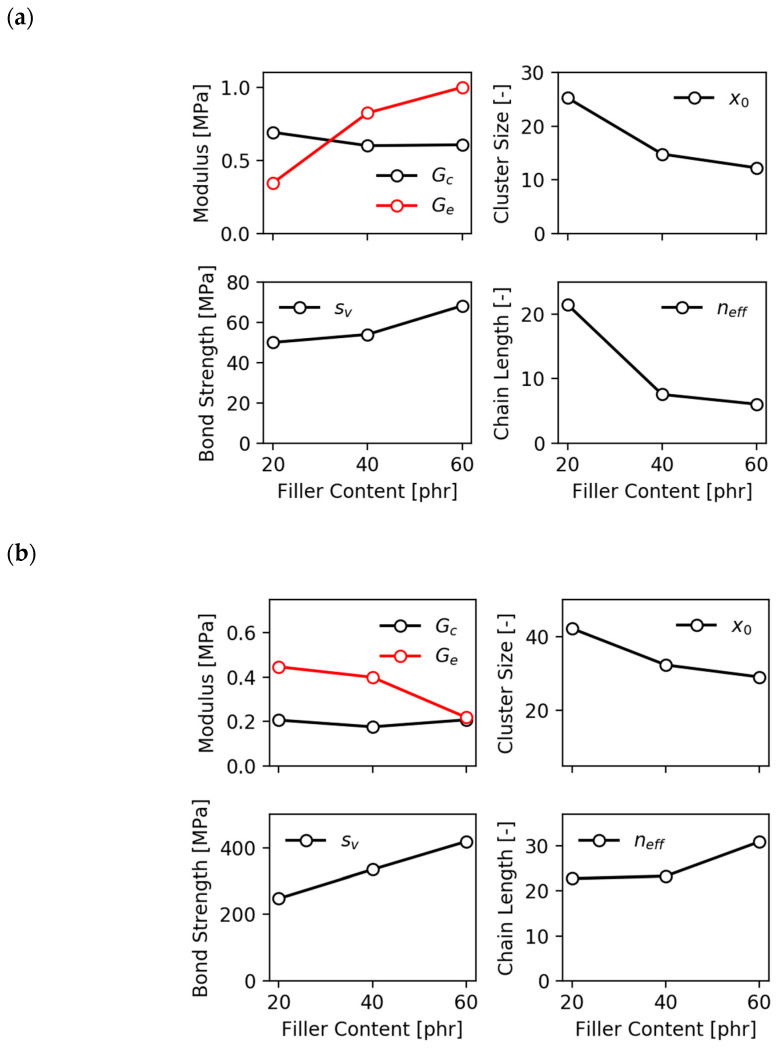
Fitting parameters obtained with the frame independent model of stress softening of the EPDM/2.4-PHR *N*-cyclohexyl-2-benzothiazolylsulfenamide (CBS) samples as shown in Figure 6, using (**a**) data up to 100% strain and (**b**) up to 200% strain.

Figure 6a,b shows fits of stress–strain cycles of the EPDM/CB samples with the frame-independent model (α=1) up to 100% and 200%, respectively. In both cases the stress-softening effect is reproduced fairly well, though for the 200% fit systematic deviations are seen for the sample with 60-PHR N339 in the small strain regime. The effect of filler concentration on the fitting parameters is depicted for both cases in Figure 7a,b, respectively. All values of the parameters are found in a reasonable range. However, they strongly depend on the range of the fitted stress–strain data up to 100% and 200%, respectively. This indicates that the DFM cannot simply be extended to strains up to 200% because additional mechanisms of stress softening may appear at larger strains, e.g., detachment of the polymer from the filler surface. Nevertheless, the quite reasonable fits in Figure 6b show that the simplified DFM can be used as an empirical model also for larger strains up to rupture of the samples. Due to the simplifications of the frame independent model compared to the original DFM it makes no sense to discuss the dependence of fitting parameters on filler concentration in detail. The more interesting point is the high stability of the fitting procedure with parameters that all are positive and can easily be reproduced. Most important is the ability to implement the simplified model into a finite element code for fast FEM simulations by using standard methods. This is of major interest for the rubber industry and will be a task of future work.

## 5. Conclusions

A micromechanical model of stress-softening and hysteresis of filler reinforced elastomers was presented, which is based on a non-affine tube model of rubber elasticity and a generalized three-dimensional Kantor–Webman model of flexible chain aggregates, describing the deformation and fracture of filler clusters in the stress field of the rubber matrix. This dynamic flocculation model (DFM) has been shown to reproduce the complex quasi-static deformation behavior of filler reinforced elastomers upon repeated stretching with increasing amplitude fairly well. It is described in some detail in the theoretical section, whereby two different fracture mechanisms of filler–filler bonds, denoted “monodisperse” and “hierarchical” bond fracture mechanism, are considered. In the first approach all bonds are considered to be equal (monodisperse) having the same strength. In the second approach a hierarchy of bond strengths is realized, because during cluster–cluster aggregation the mobility of the clusters decreases with cluster size.

In the experimental section the DFM is adapted to a series of aged EPDM samples which were treated in an oven at 130 °C for different thermo-oxidative aging times. The fitting parameters indicate that the crosslinking density remains almost constant while the entanglement density increases slightly. This indicates that the sulfur bridges in EPDM networks are quite stable even at 130 °C aging temperature, which can be related to the mono-sulfidic nature of the crosslinks. The observed hardening of the composites with increasing aging time is mainly attributed to the relaxation of filler–filler bonds. This results in an increased strength of the bonds, which produces a larger stiffness and filler-induced hysteresis of the composites.

The two different bond fracture mechanisms are investigated by separate adaptations to the full series of aged EPDM composites. They show that the “hierarchical” bond fracture mechanism delivers better fits and more stable fitting parameters, though the evolution of fitting parameters with aging time is similar for both models. Therefore, it is concluded that the “hierarchical” bond fracture mechanism, which takes into account that the mobility of clusters decreases with cluster size, appears to be realized in filler reinforced elastomers.

In the last section a frame-independent simplified version of the DFM is proposed that focuses on an easy implementation of the stress-softening effect of filled rubbers into a finite element codes by using standard methods. The model is shown to reproduce the stress-softening effect of EPDM samples with varying amount of carbon black fairly well. Therefore, it appears to be well suited for performing fast FEM simulations of highly filled rubber goods, where stress-softening cannot be neglected.
